# Nurse Navigator: development of a program for Brazil*

**DOI:** 10.1590/1518-8345.3258.3275

**Published:** 2020-06-01

**Authors:** Fernanda Felipe Pautasso, Thafarel Camargo Lobo, Cecília Dias Flores, Rita Catalina Aquino Caregnato

**Affiliations:** 1 Santa Casa de Misericórdia de Porto Alegre, Ambulatórios do Hospital Santa Rita, Porto Alegre, RS, Brazil.; 2 Universidade Federal de Ciências da Saúde de Porto Alegre, Porto Alegre, RS, Brazil.

**Keywords:** Patient Navigation, Nursing, Oncology, Nurses, Patient-Centered Care, Neoplasms, Navegação de Pacientes, Enfermagem, Oncologia, Enfermeiros, Cuidado Centrado no Paciente, Neoplasias, Navegación de Pacientes, Enfermería, Oncología, Enfermero, Atención Dirigida al Paciente, Neoplasia

## Abstract

**Objective:**

to develop a Navigation Program for cancer patients, based on the model proposed by The GW Cancer Institute at George Washington University, adapted to the reality of a Brazilian High Complexity Center in Oncology.

**Method:**

a convergent care research applied in the development of a patient navigation care process, based on the model proposed by George Washington University, adapted for a High Complexity Center in Oncology in Brazil. Phases of the Convergent Assistance Research: conception, instrumentation, scrutiny, analysis and interpretation. These were correlated with the stages of the Program Development Cycle. Scale designed to categorize patients into navigation levels, validated by the Delphi Technique, with 12 specialists.

**Results:**

in the diagnosis, patients with head and neck cancer were defined for inclusion in the Navigation Program. Planning and implementation took place simultaneously, allowing the basic formatting of the program and its processes to be designed. Navigation Needs Assessment Scale designed to select the patient to join the Program and determine the recommended support. The scale validation had a consensus index of 96.42%. Evaluation of the stages of the cycle occurred through the adapted Plan/Do/Check/Act cycle.

**Conclusion:**

a Navigation Program was developed adapted to the Brazilian reality, and attributions of the navigators were created.

## Abstract (pt)

**Objetivo:**

desenvolver um Programa de Navegação para pacientes oncológicos, fundamentado no modelo proposto pelo The GW Cancer Institute da George Washington University, adaptado à realidade de um Centro de Alta Complexidade em Oncologia brasileiro.

**Método:**

pesquisa convergente assistencial aplicada no desenvolvimento de um processo assistencial de Navegação de Pacientes, fundamentado no modelo proposto pela George Washington University, adaptado para um Centro de Alta Complexidade em Oncologia no Brasil. Fases da Pesquisa Convergente assistencial: concepção, instrumentação, perscrutação, análise e interpretação. Essas foram correlacionadas com as etapas do Ciclo de Desenvolvimento de Programas. Idealizada Escala para categorizar os pacientes em níveis de navegação, validada pela Técnica de Delphi, com 12 especialistas.

**Resultados:**

no diagnóstico definiu-se pacientes com câncer de cabeça e pescoço para inserção no Programa de Navegação. Planejamento e implantação ocorreram simultaneamente, permitindo realizar o desenho da formatação básica do programa e seus processos. Escala de Avaliação de Necessidade de Navegação elaborada com a finalidade de selecionar o paciente para ingressar no Programa e determinar o suporte recomendado. A validação escala teve índice de consenso de 96,42%. Avaliação das etapas do ciclo ocorreram através do ciclo *Plan/Do/Check/Act* adaptado.

**Conclusão:**

um Programa de Navegação adaptado à realidade brasileira foi desenvolvido, bem como, as atribuições dos navegadores.

## Abstract (es)

**Objetivo:**

desarrollar un Programa de Navegación para pacientes con cáncer, basado en el modelo propuesto por The GW Cancer Institute de la Universidad George Washington, adaptado a la realidad de un Centro de Alta Complejidad en Oncología brasileño.

**Método:**

investigación de atención convergente aplicada en el desarrollo de un proceso de atención de navegación para pacientes, basado en el modelo propuesto por la Universidad George Washington, adaptado para un Centro de Alta Complejidad en Oncología en Brasil. Fases de la investigación de asistencia convergente: concepción, instrumentación, escrutinio, análisis e interpretación. Estos se correlacionaron con las etapas del ciclo de desarrollo del programa. Escala diseñada para clasificar a los pacientes en niveles de navegación, validado por la Técnica Delphi, con 12 especialistas.

**Resultados:**

en el diagnóstico, los pacientes con cáncer de cabeza y cuello fueron definidos para su inclusión en el Programa de Navegación. La planificación y la implementación se llevaron a cabo simultáneamente, permitiendo diseñar el formato básico del programa y sus procesos. Escala de evaluación de necesidades de navegación diseñada para seleccionar al paciente que se unirá al Programa y determinar el apoyo recomendado. La validación de la escala tuvo un índice de consenso del 96,42%. La evaluación de las etapas del ciclo ocurrió a través del ciclo adaptado *Plan/Do/Check/Act*.

**Conclusión:**

se desarrolló un programa de navegación adaptado a la realidad brasileña, y fueron creadas asignaciones de los navegadores.

## Introduction

Approximately two-thirds of the global cancer deaths occur in developing countries, where mortality rates are highest due to late diagnosis and difficulty in accessing treatments^(^ 1 ^)^. According to the National Cancer Institute (Instituto Nacional de Câncer, INCA), the body that provides epidemiological information regarding cancer in Brazil, it was estimated for the country in the 2018-2019 biennium, the occurrence of approximately 600 thousand new cases of cancer^(^ 2 ^)^. Globalization, urbanization and increased life expectancy are data that can explain these estimates^(^ 3 ^-^ 4 ^)^.

In Brazil, since the 1990s, the Ministry of Health (MoH) has invested efforts to face the growing demand for cancer treatment in the country^(^ 5 ^-^ 6 ^)^ in a more organized and effective way. In this sense, currently, within the scope of the Brazilian Public Health System (Sistema Único de Saúde, SUS), among the main measures already in place are the registration and organization of a hierarchical network of establishments defined as High Complexity Centers in Oncology (Centros de Alta Complexidade em Oncologia, CACONs) to offer assistance specialized and integral in the area, until the current National Policy for the Prevention and Control of Cancer in the Health Care Network of People with Chronic Diseases^(^ 5 ^-^ 6 ^)^.

In supplementary health, aiming at the reorganization of the oncology care network, the National Supplementary Health Agency (Agência Nacional de Saúde Suplementar, ANS) launched in 2016 the OncoRede Project, whose proposal is to articulate a care network, restructuring the diagnosis process, improving screening and measuring strategies impacts the actions on the performance of the supplementary health system^(^ 3 ^)^. Pointing out several strategies, contextualized and based on evidence from those already implemented and widely used in other countries to carry out this reorganization, it proposes the structuring of an organized Cancer Care Model in Supplementary Health^(^ 3 ^)^. To achieve this goal, they suggest, among other measures, the implementation of patient navigation programs for oncology in Brazil, with the role of the patient navigator figure, named as “Care Assistant”^(^ 3 ^)^. It indicates for the performance of this function the nurse for his knowledge, training and area of action^(^ 3 ^)^.

Patient Navigation (PN) is a process in which an individual, called a patient navigator, guides people diagnosed or suspected of having a chronic disease, helping them to “navigate” through the health system and services^(^ 7 ^-^ 8 ^)^. It is performed by a patient navigator, involving a series of actions that lead to a certain objective (for example: assistance in a timely manner through the elimination of barriers to access assistance). In this context, a navigation program is a fusion between the navigation process - navigators - actions, which comprise the assistance and administrative processes of a given service and health system, designed and adapted to the profile of the assisted patients. It is a widely promoted approach to increase the likelihood that patients will have an effective adherence to the recommended treatment, reducing socioeconomic, racial and ethnic barriers to care^(^ 8 ^-^ 9 ^)^.

This concept was originally developed by the American physician Harold Freeman in partnership with the American Cancer Society (ACS) in 1990, at the Harlem Hospital in New York^(^ 8 ^-^ 10 ^)^. In this context, the first PN Program was originally designed for cancer patients, in which the navigators were volunteers (lay people and/or health professionals)^(^ 7 ^-^ 8 ^,^ 10 ^)^. There are nine theoretical principles that underpin PN established by Dr. Freeman, developed during his more than 20 years of experience, namely^(^ 7 ^-^ 9 ^)^: 1. The PN is a health service whose model is centered on the patient and its focus is to make the patient’s movement through the health system smooth and timely throughout the *continuum* of care; 2. The PN serves to facilitate the access of the patients to care through the integration of fragmented health systems, creating a continuous flow of care throughout the *continuum* of care; 3. The main function of navigation is to eliminate barriers that prevent access to health services and, in order to be effective, it is necessary to establish a close relationship between patient and navigator; 4. The scope of PN programs must be clear and well defined in relation to their practice and what distinguishes the roles and responsibilities, therefore, navigators must be integrated into the multi-professional team; 5. The delivery of the navigation service must be cost-effective and proportional to the training and skills necessary to navigate the patients; 6. The determination of which type of navigator will carry out the process must be based on the level of knowledge and skills necessary for each phase of the care trajectory of the patients. 7. It is essential to determine at what point of assistance the navigation should start and when it should be finished; 8. The navigation process must provide the connection of disconnected health systems; 9. The PN system needs coordination^(^ 7 ^-^ 9 ^)^.

PN is constantly evolving and programs, nowadays, have also been targeted at patients with other chronic diseases^(^ 11 ^-^ 12 ^)^. This process is also implemented in primary health care in countries like Canada and the United States of America (USA) for patients with diseases such as heart failure, chronic arterial hypertension and type 2 diabetes^(^ 13 ^-^ 14 ^)^. Currently, in international programs, navigators are health professionals, students and lay volunteers, each with specific duties according to their level of knowledge^(^ 13 ^-^ 14 ^)^. In countries like the USA, there is no consensus on the previous academic training of navigators, however in Canada and Australia most navigators are nurses^(^ 3 ^,^ 15 ^-^ 16 ^)^.

The Navigator Nurses (NNs) emerged to assist cancer patients from the first Navigation Program (NP)^(^ 17 ^-^ 18 ^)^. These professionals use their specialized knowledge, clinical experience and skills to provide patients with care focused on the physical, social and emotional aspects^(^ 12 ^)^. They direct and guide patients, families and caregivers for joint decision-making with a multidisciplinary team responsible for treatment^(^ 18 ^)^. The actions developed by these professionals go beyond the management of care^(^ 17 ^-^ 18 ^)^. They supervise the entire treatment process, empowering patients, providing information and support, acting as a link between them and the team professionals^(^ 17 ^-^ 18 ^)^.

The PN is considered an important differential in oncology services in Brazil, mainly with the role of the navigator nurse^(^ 19 ^-^ 20 ^)^. In addition to acting as a care coordinator, this professional contributes to patient care by providing the necessary support to overcome the impact of diagnosis and treatment, helping to overcome the main barriers that hinder access to services and health systems^(^ 12 ^,^ 18 ^,^ 20 ^)^.

Few health institutions in Brazil have this type of program in place and, in existing locations, the service is aimed at patients with breast cancer and navigation is performed by social workers and/or nurses^(^ 19 ^)^. However, the figure of the NN, with its attributions, specificities and the importance of the role it plays at the international level, has not yet been the subject of studies and/or publications in the country^(^ 19 ^)^.

In this sense, one of the authors, for acting in a reference hospital in oncology recognized as a High Complexity Center in Oncology (CACON) and by appropriating the concepts and practices that constitute the navigation of patients, and the operation of such programs, established the purpose of developing this type of Program for that location. The guiding question of this study was the following: as a Patient Navigation Program for cancer patients, based on the model proposed by The GW Cancer Institute of the George Washington University, it will be able to meet the reality existing in a CACON?

Thus, developing a Navigation Program for cancer patients, based on the model proposed by The GW Cancer Institute at George Washington University, adapted to the reality of a Brazilian CACON was the objective of this research.

## Method

It is a convergent care research (Pesquisa Convergente Assistencial, PCA), a methodology that seeks to provide the participatory insertion of the researcher in the field of care practice while being involved with the objectives of the research^(^ 20 ^)^. It is developed through the following phases: conception, instrumentation, scrutiny, analysis and interpretation^(^ 20 ^)^. The PCA, as it has a dynamic and integrated nature of assistance, is an investigative and innovative method that allows the exploration, reflection and deepening of different themes in health^(^ 21 ^-^ 22 ^)^. In this sense, it represents a challenge insofar as it seeks to impress changes and technological innovations in the instituted health space^(^ 22 ^-^ 23 ^)^.

**Figure 1:**
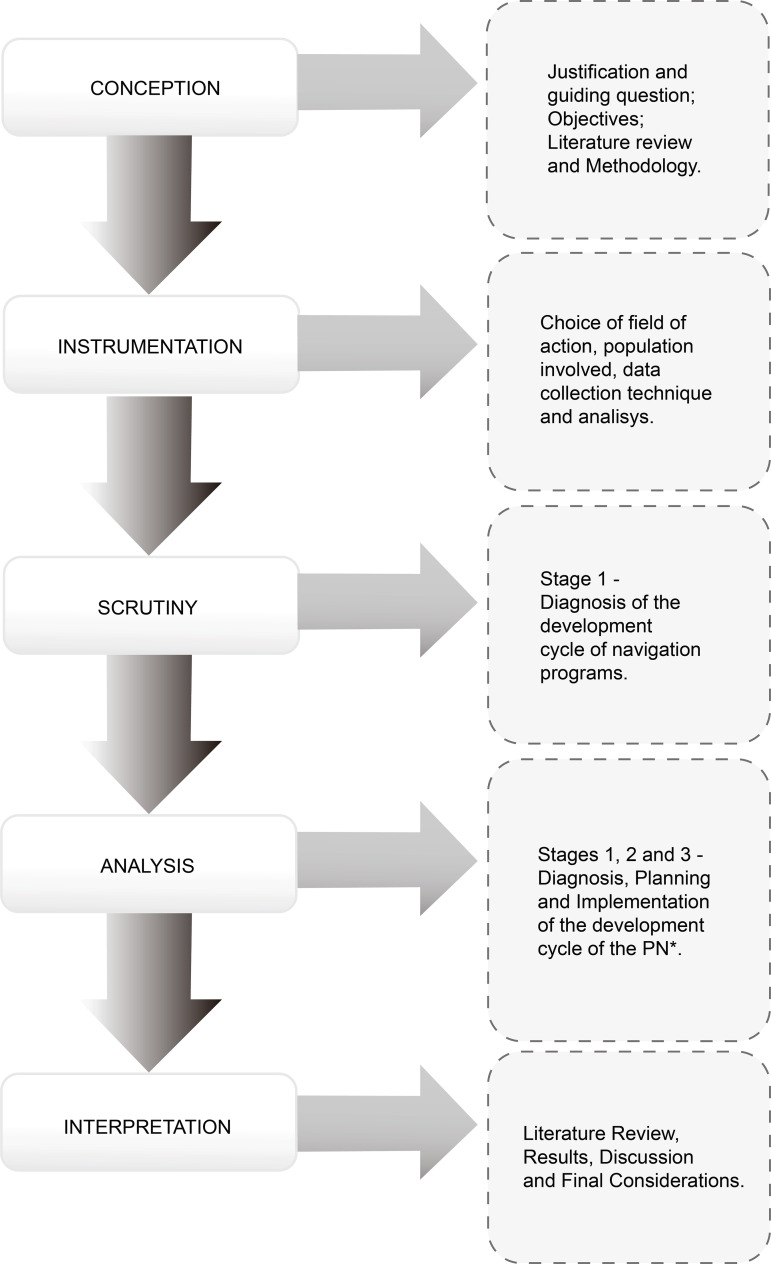
Organization of the research stages according to Convergent Care Research and the relationship with the Development Cycle of the NP*. Porto Alegre, RS, Brazil, 2018 ^*^NP = Navigation Program

This study included the development of a new assistance process through the PN, based on the adapted method and developed in the Executive Training on Navigation and Survivorship: Finding Your Patient Focus do The George Washington University (GW) Cancer Institute’s Center for the Advancement of Cancer Survivorship, Navigation and Policy (caSNP), of the George Washington University (USA) conducted in e-learning format by one of the authors. The theoretical-philosophical basis that supported the development of this study corroborated with the same that was used by the University in the elaboration of the course, the concept and the principles of patient navigation conceived by the American doctor Harold Freeman. As recommended by The GW Cancer Institute, NP program planning must be structured based on the Program Development Cycle, which has four stages (diagnosis, planning, implementation and assessment) and were adapted by the researcher to adapt them to the reality in that CACON. The correlation between the phases of the PCA’s methodological path and the Program Development Cycle is shown in Figure 1.

The first phase, “Conception”, contemplated the initial research definitions, such as: choice of theme; definition of the guiding question; determination of research objectives; literature review and the choice of philosophical theoretical foundation to guide the new care process.

In the “Instrumentation”, second phase, the field of action was defined, the population involved in the study and the data collection and analysis technique, the PN Program Development Cycle, was chosen.

In the “Scrutiny” phase, the diagnosis stage took place, so that this was possible, data collection took place in the electronic medical records system and in the existing management reports of the institution.

In the “Analysis”, the process called apprehension occurred, where an organization of the data obtained in the diagnosis was carried out and was completed in the second and third stages of the cycle (planning and implantation). Each assistance and administrative process was observed (registered in Excel spreadsheets) and through the development of the practice and interaction with the professionals in the service, the basic formatting of the navigation program and its processes was created.

In the last phase of the PCA, the “Interpretation”, the processes of synthesis, theorization and recontextualization took place. In the first two processes, subjective data analysis was performed and the relationship between the information collected and the philosophical theoretical foundation used in the study was established. The last was to give meaning to the results obtained and in the socialization of them. The data were analyzed in the evaluation stage, through the application of the adapted PDSA cycle. All were operationalized during the literature review, the second and third stages of the cycle.

The field of action of this study was the Brazilian Public Health System (SUS) Outpatient Clinic of a hospital classified as a High Complexity Center in Oncology (CACON), a private institution of a philanthropic character, located in the south of Brazil. It provides assistance in oncology being a national reference in the area, acting in the prevention, diagnosis and treatment for clients referred by the SUS, private or through agreements.

The target population selected for this study was an intentional sample, consisting of health professionals, fourteen nurses (seven area managers and seven assistants), four doctors (two clinical oncologists, a head and neck surgeon and a palliative care), a psychologist and a speech therapist who work at the study institution and a nursing professor.

This study followed the current legislation, according to the terms of Resolution 466/2012, of the National Health Council and was submitted to the Ethics and Research Committee of the University and the hospital, having been approved with CAEE No. 67250617 0 0000 5335. All the participants were informed and provided the consent form.

In order to assess patients in relation to their need for navigation, it was necessary to create a Need for Navigation Assessment Scale (Escala de Avaliação de Necessidade de Navegação, EANN). This instrument aims to provide opportunities, based on categories and biopsychosocial criteria, to classify patients in relation to the need for navigation, for inclusion or not in the PN program developed for the CACON of the study. As no instrument was found in the literature for this purpose, after its construction, which occurred during the planning and implementation stages, EANN was validated using the Delphi Technique (DT).

Defined as a systematic methodology for judging information, DT is considered a research tool that seeks a consensus of opinions from a group of experts on a given topic, through validations articulated stages or cycles^(^ 24 ^-^ 25 ^)^. It is intended for situations where there is no and/or a lack of historical data and, in the field of nursing, it has been adopted for the validation of conduct and diagnostics^(^ 23 ^-^ 25 ^)^.

For the validation of the instrument, a panel of experts was selected, consisting of 21 health professionals with technical knowledge and experience in oncology. The cut-off points for obtaining a consensus was set at 80%, since it is not recommended in the literature that, in situations of production scarcity, obtaining consensus with less than 75% percentiles^(^ 25 ^)^. In the first round of DT, of the invited specialists, 17 participated and in the second, 12 professionals responded to the survey.

## Results

During the PN Program Development Cycle, in the diagnosis stage started in July 2017, an initial assessment was carried out in order to establish the demographic profile of the patients seen at the service. A total of 7,310 patients were seen, from January to June 2017, of which 56.30% were women, most of whom were aged between 61 - 75 years old (43%) and were married (45.88%). As for the place of origin, the greatest demand for assisted patients came from Porto Alegre and the metropolitan area, making a total of 61.46%, with the rest coming from other places in Rio Grande do Sul. Most of the individuals had incomplete elementary education totaling 40.36%, and only 4.99% had complete higher education.

Regarding the functioning profile and the services provided in the sector, the specialty with the highest number was clinical oncology with a total of 7,308 consultations (45.17%). This expressive number is justified by the fact that this specialty assists patients with all types of cancer, for the definition and referral of the clinical treatment of the disease. Breast surgery (1,893 patients) and head and neck surgery (1,574 patients) constituted the second and third specialty with the highest volume of consultations for a specific type of neoplasia.

At the end of this phase, which took place in September 2017, the definition of the key points needed to start planning the Program was carried out, based on the following questions: What are the main barriers faced by patients when accessing the service? What will be the population included in the program and how will they be assessed regarding their real need for navigation? Which navigation model will be developed? What will be the objectives of the navigation program and the desired outcomes?

The planning stage took place simultaneously with the realization of a navigation pilot during the implementation phase, since the design of its basic format and its processes was built during observation and development in practice with patients.

**Figure 2:**
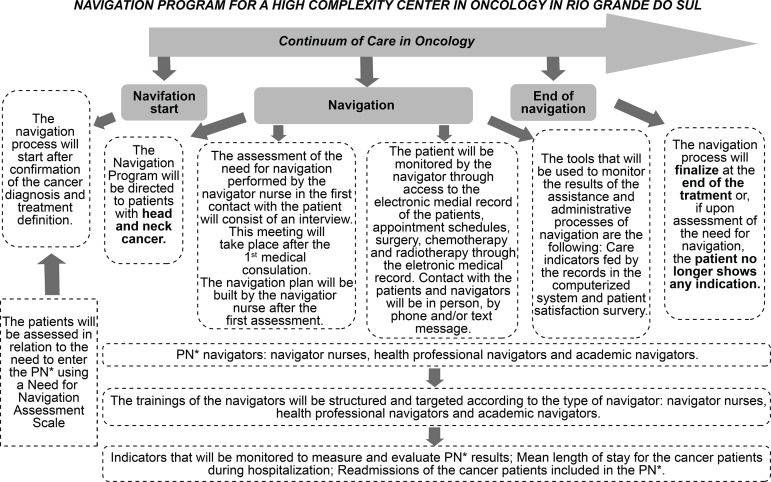
Basic structure of the Navigation Program developed for the High Complexity Center in Oncology of this study. Porto Alegre, RS, Brazil, 2018 ^*^NP = Navigation Program

Based on the definition of the fundamental points and based on the information collected and presented previously, the Basic Structure of the Program was created, designed to guide its functioning, as shown in Figure 2.

Thus, the choice of the target population, patients with head and neck cancer, was made based on the analysis of the data referring to the profile of care, functioning profile and assistance profile of the field of action.

In the implementation stage, the implementation of the navigation pilot for structuring the program model for CACON was carried out in five phases and occurred together with the planning stage, with the participation of one of the professionals involved in the research. During patient care, the researcher and one of the nurses participating in the research used interview instruments built for this stage.

At this moment, the first version of the Navigation Needs Assessment Scale (Escala de Avaliação de Necessidade de Navegação, EANN) was built, since not all patients had the same needs in relation to the navigation. This instrument was designed to categorize patients in terms of navigation levels, guided by biopsychosocial and cultural categories and criteria identified as points of greater or lesser need for patient navigation, whose identification occurs from the first interviews.

The main objective of the EANN is to establish whether the evaluated patients have a real need to enter an NP and what support is recommended. The instrument is based on the answers provided by the patients at the time of the interviews with the navigator. It should be applied, initially, in the first interview conducted, in order to determine the needs and to base the elaboration of the navigation plan, which will be elaborated by the navigator nurse. The key questions of the EANN should be directed to patients and adapted in a format that allows understanding of the question, so that the answer is suitable for evaluation.

The validation of this instrument using TD was performed in two rounds. For that, the choice of the components of the panel of experts was made intentionally, related to their knowledge and experience in the area of oncology. Thus, 21 professionals were selected, of which 17 accepted the invitation, signing the FICF and participating in the first round. Five of these did not participate in the second round, leaving a total of 12 specialists, and a consensus index of 96.42%, with the final version of the scale validated (Figure 3).

**Figure 3:** Final version of the Navigation Needs Assessment Scale. Porto Alegre, RS, Brazil, 2018

Navigation needs assessmen scale (Escala de avaliação de necessidades de navegação, EANN*)					
Category	key questionto the patients		Criteria		Score
Patient's understanding regarding the diagnosis	What did your doctor tell you about your health problem?		Uderstands your diagnosis		1
	If yes, what did he/she tell you?		Partly understands the diagnosis		2
	From all that said, what did you understand?		The patient did not understand an anything or whats was said about his/her diagnosis		3
Communication ability	Observe the patient's ability to communicate during the ir responses to the questions:		No communication difficulties		1
	Is there any physical barrier that makes it impossible?		Has some communication difficulty		2
	Are there language, cognitive or cultural barriers that partially or completely hinder their ability to communicate?		Cannot communicate		3
Understanding the treatment trajectory	Did your doctor tell you about how your treatment will be? If so, what did he tell you?		Understands the treatment trajectory well		1
	Did you undertand what you were told? If so, what did you undertand?		Partially understands the treatment trajectory		2
	Do you know what the stages of your treatment will be and how they will be carried out?		Does not understand the treatment trajectory		3
Organizational ability to perform the treatment	Do you have any difficulties to come for the appointments, treatments, and/or perform exams? If so, which?		The patient is able to organize himself to attend to appoiments, treatments and/or to carry out necessary exams		1
	Would you like to receive help from someone to organize your appointment, treatment and exam schedules? If so, how could this person help?		The patient has difficulty organizing himself to attend to appointments, treatments and/or to carry out necessary exams and needs help to do so		2
Access to health services/system (transport conditions, commuting between services necessary for treatment within and outside the health institution)	Do you have any difficulty going to the hospital and/or another location (outpatient clinics, diagnostic imaging centers, laboratories, other hospitals) to carry out your treatment? If so, which one(s)?		It is easy to access the service by means of transport (public or private) and knows how to locate the place (hospital or other service) of your treatment		1
	Do you know where the hospital is and the sectors where you do your treatment? If so, where?				
	Do you use any transport (public or private) to come to the hospital? If so, Which onde?		It is easy to access the transport (public or private) to go to the health service and difficulty in locating the hospital/service of your treatment (other reasons)		2
	Do you have the means to go the hospital and/or other places to caarry out your treatment? If so, how do you get there?		Difficulty in accessing a transport (public or private) to go to the hospital/service for tratment and difficulty to go the place (hospital/sector) for treatment		3
Family sypport	Are you accompanied by a family member and/or caregiver when you come to consultations and/or carry out your treatment? If so, who?		There is full support and monitoring: the family/caregiver participates in decisions and care and accompanies the patient always of treatment		1
	Do you have any family, friend and/or caregiver support you during the treatment? If so, who?		There is partial support and monitoring: the family/caregiver participates in decisions and care and accompanies the patient in some moments of treatment		2
	Who do you talk to, besiders hospital/service professionals, about your health problem, treatment and the changes that are occurring?		Absence of support: the patient has no family member/caregiver who participates and monitors their treatment		3

Total Score:___________________________________					
**Navigation Level 1:** Navigation performed by an academic navigator and a professional navigator most of the time, with support from the nurse navigator.		Key MINIMUM SCORE: 6 POINTS MAXIMUM SCORE: 17 POINTS		6 TO 9 POINTS: NO NAVIGATION NEEDED	
				10 TO 12 POINTS: NAVIGATION NEED LEVEL 1	
**Navigation Level 2:** Navigation performed by the nurse navigator, mainly with other navigators.				13 TO 12 POINTS: NAVIGATION NEED LEVEL 2	

In this stage, the basic assignments of the navigators, the profile of the nurse navigator and the professional navigators were also structured at this stage, based on the knowledge and skills necessary to perform the function, and the qualifications for their qualification, considering the necessary knowledge areas for its performance, as shown below, in Figures 4 and 5.

**Figure 4:** Basic assignments of navigators. Porto Alegre, RS, Brazil, 2017

List of Assignments
Help patients to identify and overcome challenges to obtain quality health care.
Help patients to access care and navigate the health system.
Assist patients to mitigate and overcome barriers to obtain care.
Assess the main barriers to care, involving patients and family members/caregivers in the definition of solutions to overcome them.
Identify the necessary resources to meet the needs of patients (biopsychosocial and spiritual), considering social, cultural and cognitive conditions, making the necessary referrals with the multidisciplinary team.
Educate patients and caregivers about cancer treatment, the roles of multidisciplinary team members and what to expect from the health system and service.
Contribute to the development, implementation and evaluation of the patient navigation program.
Encourage communication between patients, family members/caregivers and professionals responsible for health care to favor and optimize results.

**Figure 5:** Profile of the Navigator Nurse. Porto Alegre, RS, Brazil, 2017

Dimension	Assignments
Care Coordination	Assess patients for their need for navigation from the EANN*.
	Develop and implement the navigation plan for patients included in the NP^†^.
	Identify possible barriers to obtaining care and facilitate access to the services and resources needed to mitigate them.
	Promote and implement a consistent and comprehensive navigation plan, using appropriate tools and methods for assessment, based on the best scientific evidence.
	Participate in defining the care plan with the multidisciplinary team and patient.
	Coordinate the care plan with the team, accompanying the patient during their treatment and providing support through guidance, health education.
	Facilitate the promotion of individualized care considering the physical, cultural, biopsychosocial and spiritual needs for patients and family members/caregivers.
	Assist patients to overcome barriers related to treatment goals, palliative care and end of life concerns through an ethical and humanized approach.
	Know health systems and the impact of their processes for treatment in a timely manner, providing support to patients and favoring safe decision-making in conjunction with the multidisciplinary team.
	Provide support to patients for the organization of appointments, exams and other procedures necessary for their treatment, aiming to promote their adherence and participation in planning.
	Assist and make it possible for patients to attend consultations and other tests and procedures necessary for treatment.
	Coordinate the operation of the NP^†^ and performance of the navigator team.
Leadership	Supervise the execution of navigation processes.
	Evaluate the results and outcomes related to the NP^†^.
	Implement improvements and/or new processes to improve the quality of the NP^†^.
	Develop tools to optimize NP results^†^.
	Act as a link between patients, their families/caregivers and the care team, favoring the strengthening of the bond between them.
Communication	Promote effective communication between the multidisciplinary team and patients.
	Work with the multidisciplinary team to promote patient-centered care that includes shared decision-making, setting goals related to treatment and evaluating outcomes.
	Favor and direct access to psychological and/or social support according to the needs of patients and family members/caregivers throughout the treatment trajectory.
	Ensure that communication is culturally appropriate for the level of understanding and cognition of patients and family members.
	Empower patients through the development of a personalized educational plan, aimed at promoting patients' autonomy in relation to their treatment.
Health education	Develop an educational plan for patients and family members/caregivers considering possible and existing barriers to care.
	Promote health education for patients, families and caregivers on diagnosis, treatment, management of side effects and other care to prevent the occurrence of complications.
	Provide health education and personalized support, favoring patients' autonomy in decision making regarding their treatment.
	Give to patients and family members/caregivers information based on the best scientific evidence to answer questions about treatment and potential expected results.
	Provide information aimed at promoting quality of life during treatment, guiding you on the importance of maintaining a healthy and self-care lifestyle.
	Promote and favor adherence of the patients to treatment through health education.
	Guide and inform patients and families/caregivers about the health system, access to available resources and services, about the roles of members of the multidisciplinary team.
Guidance and Information	Guide and inform patients about times of procedures, consultations, exams and necessary accompaniments for their treatment.
	Guide patients on care and management of possible complications related to their treatment.
	Provide access to information on the assistance needed according to the needs of patients.
	Inform patients about their rights and duties in relation to their treatment and diagnosis.
	Direct patients to the necessary services for the proper progress and continuity of their treatment.

* EANN = Navigation Needs Assessment Scale;

† NP = Navigation Program

In the evaluation, the last stage of the NP development cycle, the analysis of stages 1, 2 and 3 of the program development cycle were evaluated through the PDSA cycle adapted by the researcher (*Plan* - *Do - Study* - *Act* or continuous improvement cycle). This is a quality management tool that establishes the evolution of the system through the continuous learning of people and organizations resulting in innovation and improvement of products and processes^(^ 26 ^)^. Each of them was analyzed in terms of meeting the objectives and obtaining the expected results and all reached the established goals.

## Discussion

The NP is a process that involves a series of actions necessary to achieve a certain outcome/objective^(^ 8 ^,^ 19 ^,^ 27 ^)^. In this perspective, a NP program consists of formatting this process to meet the needs of patients assisted in a given health service, whose actions involving the assistance and administrative routines of the place for which it is designed, are carried out by the navigators. Its operating structure, in order to be adequate and directed to achieve the desired outcomes, needs to be planned in a detailed way and as personalized as possible, as the model of one institution will not always meet the peculiarities of another.

Thus, in order to meet the main premise of this study “to develop an NP Program appropriate to the reality of CACON” and within the context of the Brazilian health system, it was necessary to study the care provided to cancer patients and their particularities, especially regarding this patient is already in the tertiary complexity service. To this end, the methodology proposed by the GW Cancer Institute for the development of navigation programs, includes a cycle with 4 stages where, based on the diagnosis of the needs of the service, health system and patients (stage 1 - diagnosis), a personalized program is designed (stage 2 - planning) followed by its implementation (stage 3 - implantation) and continuous evaluation (stage 4 - evaluation)^(^ 28 ^)^. These stages were strictly followed for the development of the NP Program, being adapted to meet the Brazilian reality of a CACON, since the model used in the rationale is American.

The consulted literature suggests that NP is more effective, when directed at patients with barriers to care, and can be identified through an assessment of the social determinants of health^(^ 29 ^)^. Therefore, it is recommended that the services analyze their populations to determine which patients need navigation before implementing a program^(^ 29 ^-^ 31 ^)^. Thus, in the first stage of this research, it was possible to view the profile of assisted patients and the functioning of the service, which has a significant volume of consultations of 7,310 consultations in 6 months, being the greatest demand originating in the capital and metropolitan region. Regarding the profile of care, the specialty that showed the largest number was clinical oncology, for assisting patients for clinical treatment of other types of cancer, followed by breast surgery and head and neck surgery. In the state of RS, due to the population and epidemiological profile, breast cancer is the first most frequent (73.07/100 thousand cases) and head and neck cancers, larynx, mouth and esophageal cancers occupy the seventh, the sixth and fifth position in frequency^(^ 2 ^)^. In this context, the program model directed at reality of the CACON was designed based on the profile of patients assisted in the services and in their functioning.

The basic structure developed for the Navigation Program developed for CACON contemplates the development of navigation processes along with a *continuum* of oncology care. Each navigation model is outlined by the type of navigator active, when the assistance will start and end the process and structured, according to the population to be assisted, being able to be directed to only one type of cancer or not, be adaptable to different social, cultural and economic realities of the service^(^ 7 ^,^ 28 ^)^. In this perspective, the beginning of the navigation of patients in the program was established to happen shortly after the confirmation of the diagnosis and definition of the treatment since, during the unfolding of the stages of the cycle, the reaction of the patients when they received the news of the pathology and about the indicated therapy, because, besides being assimilating the impact of the diagnosis itself, they were confused and lost in relation to the next steps to follow to start it. The different therapeutic modalities usually employed, such as surgical treatment, for the total or partial removal of the tumor or affected tissue, chemotherapy, where antineoplastic medications are administered on a regular basis, and radiotherapy, with direct irradiation of the site or region affected, demand an expressive amount of information that is usually released to patients and their families in this first moment and are hardly assimilated by them^(^ 4 ^,^ 32 ^)^. As a result, the barriers to access the services and exams required at this stage became evident from this moment on.

It was observed during planning and implantation that patients had different difficulties and deficiencies, regardless of their socioeconomic status. Psychosocial, economic and cultural aspects represent factors of great impact on the population’s access to recommended cancer treatment and timely care^(^ 29 ^)^. From this observation, it was felt the need to develop a scale aimed at determining which patients should be assisted in the CACON Navigation Program, stipulating that they would be assessed in relation to their need for navigation through the application of the EANN, elaborated and validated during this study. Thus, EANN offers a selection, signaling those who need to enter the program, categorizing them into more or less intense levels of navigation and contributing to the viability of the NP in relation to the physical and financial resources of the service.

Based on the data obtained during the survey of the care profile of the oncology specialties active in the field of action and the implementation developed with the special outpatient clinic for multidisciplinary care in clinical oncology, it was determined that the navigation program would be directed to a specific type of cancer given the expressive volume of consultations (average of 2,690/month) that presents the site *versus* the restricted number of possible navigators available on the service. The format and scope of the NP, together with the roles and responsibilities of its navigators, must reflect the needs of patients, the community and the health institution, for which it is designed, and the service conditions and service functioning must be adapted^(^ 28 ^,^ 33 ^-^ 34 ^)^.

The patients selected to take advantage of the developed NP are the head and neck cancer (HNC) patients that occupy the sixth position, worldwide, representing about 3% of all the neoplasms^(^ 35 ^)^. The location of this disease ends up imposing physical, social and psychological suffering on the patient and his family, due to the changes caused in the individual’s basic functions, such as food, breathing and speech^(^ 36 ^-^ 39 ^)^. The effective management of cancer treatment, particularly those with HNC, represents a substantial challenge for health systems^(^ 36 ^-^ 39 ^)^.

The first assessment of the need for navigation and the construction of the navigation plan was established as a specific function of the NN. This professional, due to his knowledge and his ability to interact with the interdisciplinary team, is able to assess patients who need more support and/or more urgent care^(^ 27 ^,^ 40 ^-^ 41 ^)^. Thus, among the benefits of the nurse in the role of navigator is the certainty of patient-centered care and effective care management in all phases of the *continuum* ^(^ 27 ^,^ 40 ^-^ 41 ^)^. In this context, the implementation of Navigation Programs, with nurses as the main actor in the coordination of care in the *continuum* of care, ensures patients, services and the health system a differential in relation to the quality of oncology care^(^ 13 ^-^ 14 ^,^ 19 ^)^.

The Program Model elaborated foresees the performance of three types of navigators (the NN, health professional navigators and academic navigators) in order to form a team under the coordination of the NN. At both levels of navigation, the performance of everyone will occur, what differs is the performance with greater or lesser intensity of the NN. The definition of these three types is due to the fact that the CACON under study is a teaching hospital, including a Multi-professional Health Residency Program (Residência Multiprofissional em Saúde, RMS), with the training and specialization of health professionals, integrating teaching-service. This integration is understood as the collective and combined work of students, residents and teachers of various training courses, with workers who are part of the health care teams of health institutions, aiming at the integrality of individual and collective care^(^ 42 ^)^.

The qualification of the navigators will be carried out in a way directed to each one of the three types, contemplating the knowledge of the oncology care practice and administrative procedures and routines, to bring about a better understanding of the context in which the patients are inserted and subsidize their health education and family members/caregivers. There is currently no evidence in the literature that reports a pattern for the level of training, indicated for the success of the performance of the patient navigator^(^ 29 ^,^ 43 ^)^.

It was established that the navigators will guide the treatment of the patient trajectory and perform care management by monitoring the records in the TASY system (appointment schedules, exams, chemotherapy and radiotherapy; patient movement; records in the electronic medical record). Communication between patients and navigators will be carried out by phone, text messages, messages from the WhatsApp app and in person, with prior appointment and/or need signaled by the assistance team and/or by the patient and family. The benefits of effective communication between patients and health care professionals are multiple, promoting the general well-being of both^(^ 44 ^)^. Effective dialog positively influences the recovery of the patient, helping to control pain, adhere to treatment, cope with the disease and improve the quality of life of individuals navigated^(^ 44 ^)^.

The indicators initially defined to monitor and analyze the impact of the navigation processes and of the entire program for the service, initially will be the average permanence of cancer patients during hospitalization; total readmissions of cancer patients included in the NP; and the satisfaction of the navigated patients. The indicators can and should be used to analyze the effectiveness of the NP, improve its effectiveness and generate data that support future changes in the processes, always seeking to contemplate the objectives outlined for it^(^ 44 ^)^. The evaluation of the outcomes related to navigation is a fundamental part of the program development cycle and, in this sense, the indicators enable their monitoring and analysis^(^ 45 ^)^.

It is believed that the implementation of these programs in the setting of CACONs oncology care, will bring about important changes in their care context. In this sense, this study does not represent an end point, but rather a beginning regarding the study of navigation and the performance of the actors in this process, especially the figure of the navigator nurse, in the Brazilian context. Thus, its main contribution to the advancement of scientific knowledge is the opening of a new space for discussion and development of services for professional and assistance qualification. The NP certainly reaches the reality of cancer care in the country as a light, and if developed within the perspective of patient-centered care, it will illuminate the lives of so many people who, currently, due to the barriers of access to assistance, live in the shadow of the search for humanized care and accessible to all.

The main limitation of the study was that, due to the time required for the development and adaptation of the NP Program to the reality of CACON, it was not possible to carry out an evaluation of its effectiveness, requiring future studies regarding the benefits and limitations of its operation in the service.

## Conclusion

The development of a Navigation Program for cancer patients, resulted in the structuring of a program model suited to the needs of patients and the operation of a reference service in Brazilian oncology. The creation of the Navigation Needs Assessment Scale (EANN) was an evident need for the implementation of a cost-effective program, in the reality of cancer care in the country. This scale can be used for health services that serve SUS patients and that implement an NP for cancer patients. In addition, the necessary assignments for the navigators’ performance were elaborated, according to their profile (whether nurse, student or layperson).

## References

[1] World Health Organization (2017). Global health observatory: the data repository [Internet].

[2] Instituto Nacional de Câncer (Brasil) (2018). Estimativa 2016: incidência de câncer no Brasil [Internet].

[3] Agência Nacional de Saúde Suplementar (Brasil) (2016). Projeto OncoRede: a (re) organização da rede de atenção oncológica na
saúde suplementar [Internet].

[4] Instituto Nacional de Câncer (Brasil) (2016). Estimativa 2018: incidência de câncer no Brasil [Internet].

[5] Teixeira LA, Porto M, Habib PABB (2012). Public policies for cancer control in Brazil: elements of a
trajectory. Cad Saúde Coletiva.

[6] Silva ACC, Giardinetto ARSB (2012). Public policies in oncology: reflecting on the role of
occupational therapy. Rev Ter Ocup Univ São Paulo.

[7] Esparza A (2013). Patient navigation and the American Cancer
Society. Semin Oncol Nurs.

[8] Smith J (2014). Patient navigator's role definition [Capstone Project].

[9] Freeman HP (2012). The origin, evolution, and principles of patient
navigation. Cancer Epidemiol Biomarkers Prev.

[10] Carroll JK, Humiston SG, Meldrum SC, Salamone CM, Jean-Pierre P, Epstein RM (2010). Patients' experiences with navigation for cancer
care. Patient Educ Couns.

[11] Sullivan C, Leon JB, Sayre SS, Marbury M, Ivers M, Pencak JA (2012). Impact of navigators on completion of steps in the kidney
transplant process: a randomized, controlled trial. Clin J Am Soc Nephrol.

[12] Freeman HP, Rodriguez RL (2011). History and principles of patient navigation. Cancer.

[13] Wang ML, Gallivan L, Lemon SC, Borg A, Ramirez J, Figueroa B (2014). Navigating to health: evaluation of a community health center
patient navigation program. Prev Med Rep.

[14] Kelly E, Ivers N, Zawi R, Barnieh L, Manns B, Lorenzetti LD (2015). Patient navigators for people with chronic disease: protocol for
a systematic review and meta-analysis. BMC Sistematic Rev [Internet].

[15] Walkinshaw E (2011). Patient navigators becoming the norm in Canada. CMAJ.

[16] Cook S, Fillion L, Fitch M, Veillette AM, Matheson T, Aubin M (2013). Core areas of practice and associated competencies for nurses
working as professional cancer navigators. Can Oncol Nurs J.

[17] Cantril CA (2014). Overview of nurse navigation. Chapter 1. Oncology nurse
navigation: delivering patient-centered care across the
continuum. Oncol Nurs Soc [Internet].

[18] Chillakunnel HRS, Pai MS, Fernandes DJ (2015). Oncology nurse navigator programme: a narrative
review. Nitte Univ J Health Sci.

[19] Pautasso FF, Zelmanowicz AM, Flores CD, Caregnato RCA (2018). Role of the nurse navigator: integrative review. Rev Gaúcha Enferm.

[20] Trentini M, Paim L, Silva DMGV, Lacerda MR, Costenaro RGS (2016). Pesquisa convergente assistencial. Metodologias de pesquisa para a enfermagem e saúde.

[21] Pivoto FL, Lunardi WD, Dantos SSC, Lunardi VL (2013). Convergent-assistential research: an integrative review of
scientific nursing production. Texto Contexto Enferm.

[22] Alvim NAT (2017). Convergent care research in Nursing - opportunities for
technological innovations. Esc Anna Nery.

[23] Almeida MHM, Spínola AWP, Lancman S (2009). Delphi technique: validation of an instrument to be used by
occupation therapist in gerontology field. Rev Ter Ocup Univ.

[24] Scarparo AF, Laus AM, Azevedo ALCS, Freitas MRI, Gabriel CS, Chaves LDP (2012). Reflections on the use of Delphi technique in research in
nursing. Rev Rene.

[25] Pereira RDM, Alvim NAT (2015). Delphi technique in dialogue with nurses on acupuncture as a
proposed nursing intervention. Esc Anna Nery.

[26] Machado C, Silva AB (2012). Possibilities and limits of the cycle of continuous improvement
-PDCA as an element of learning. Rev Metrop Sustent.

[27] Oncology Nursing Society (2013). Oncology nurse navigator core competencies.

[28] The Cancer Institute (2014). Executive training on navigation and survivorship: finding your patient
focus. Guide for program development.

[29] Freund KM (2016). Implementation of evidence-based patient navigation
programs. J Acta Oncol.

[30] Krok-Schioen JL, Oliveira JM, Paskett ED (2016). Cancer care delivery and women's health: the role of patient
navigation. Front Oncol.

[31] Baik SH, Gallo LC, Wells KJ (2016). Patient navigation in breast cancer treatment and survivorship: a
systematic review. J Clin Oncol.

[32] Soffiatti NRT (2000). Nursing consultant in chemotherapy ambulatory: emphasy in nursing
health education. Cogitare Enferm.

[33] Wilcox B, Bruce SD (2010). Patient navigation: a "win-win" for all involved. Oncol Nurs Forum.

[34] Pedersen A, Hack T (2010). Pilots of oncology health care: a concept analysis of the patient
navigator role. Oncol Nurs Forum.

[35] Aquino RCA, Lima MLLT, Menezes CRCX, Rodrigues M (2015). Epidemiologic aspects of mortality from oral cancer:
understanding the risks to enable the early detection of changes in
communication. Rev CEFAC.

[36] Paula JM, Sonobe HM, Nicolussi AC, Zago MM, Sawada NO (2012). Symptoms of depression in patients with cancer of the head and
neck undergoing radiotherapy treatment: a prospective study. Rev. Latino-Am. Enfermagem.

[37] Cruz FOAM, Ferreira EB, Vasques CI, Mata LRF, Reis PED (2016). Validation of an educative manual for patients with head and neck
cancer submitted to radiation therapy. Rev Latino-Am Enfermagem.

[38] Egestad H (2013). The significance of fellow patients for head and neck cancer
patients in the radiation treatment period. Eur J Oncol Nurs [Internet].

[39] Gilbert JE, Green E, Lankshear S, Hughes E, Burkoski V, Sawka C (2011). Nurses as patient navigators in cancer diagnosis: review,
consultation and model design. Eur J Cancer Care.

[40] American Nurses Association (2012). The value of nursing care coordination. A white paper of the
American Nurses Association. [Internet]. Nurs Outlook.

[41] Vendruscolo C, Ferraz F, Prado ML, Kleba ME, Reibnitz KS (2016). Teaching-service integration and its interface in the context of
reorienting health education. Interface.

[42] The Cancer Institute (2016). Advancing the field of cancer patient navigation: a toolkit for
comprehensive cancer control professionals.

[43] Uitterhoeve R, Bensing J, Dilven E, Donders R, de Mulder P, van Achterberg T (2009). Nurse-patient communication in cancer care: does responding to
patient's cues predict patient satisfaction with
communication. Psychooncology.

[44] Colorado Department of Public Health and Environment (2017). Key performance indicators for health navigator programs.

[45] The Cancer Institute (USA) (2016). Patient navigation barriers and outcomes tool TM (PN-BOTTM) Version 1.1
Quick Start Guide.

